# Genetic diversity of *Toxoplasma gondii* in South America: occurrence, immunity, and fate of infection

**DOI:** 10.1186/s13071-023-06080-w

**Published:** 2023-12-19

**Authors:** Ramayana Morais de Medeiros Brito, Gabriella de Lima Bessa, Alexandre Lazoski Bastilho, Filipe Dantas-Torres, Valter Ferreira de Andrade-Neto, Lilian Lacerda Bueno, Ricardo Toshio Fujiwara, Luisa M. D. Magalhães

**Affiliations:** 1 Laboratory of Immunobiology and Control of Parasites, Department of Parasitology, Institute of Biological Sciences, Federal University of Minas Gerais, Belo Horizonte, Brazil; 2 Centro Universitário do Planalto Central Apparecido dos Santos, UNICEPLAC, Brasília, Brazil; 3 Laboratory of Malaria and Toxoplasmosis Biology, Department of Microbiology and Parasitology, Biosciences Centre, Federal University of Rio Grande do Norte, Natal, Brazil; 4 Aggeu Magalhães Institute, Fundação Oswaldo Cruz (Fiocruz), Recife, Brazil

**Keywords:** Atypical strains, Toxoplasmosis, Immunity, Virulence factors

## Abstract

*Toxoplasma gondii* is an intracellular parasite with a worldwide distribution. *Toxoplasma gondii* infections are of great concern for public health, and their impact is usually most severe in pregnant women and their foetuses, and in immunocompromised individuals. Displaying considerable genetic diversity, *T. gondii* strains differ widely according to geographical location, with archetypal strains predominantly found in the Northern Hemisphere and non-archetypal (atypical) strains, with highly diverse genotypes, found mainly in South America. In this review, we present an overview of the identification and distribution of non-archetypal strains of *T. gondii*. Special attention is paid to the strains that have been isolated in Brazil, their interaction with the host immunological response, and their impact on disease outcomes. The genetic differences among the strains are pivotal to the distinct immunological responses that they elicit. These differences arise from polymorphisms of key proteins released by the parasite, which represent important virulence factors. Infection with divergent non-archetypal strains can lead to unusual manifestations of the disease, even in immunocompetent individuals.

**Graphical Abstract:**

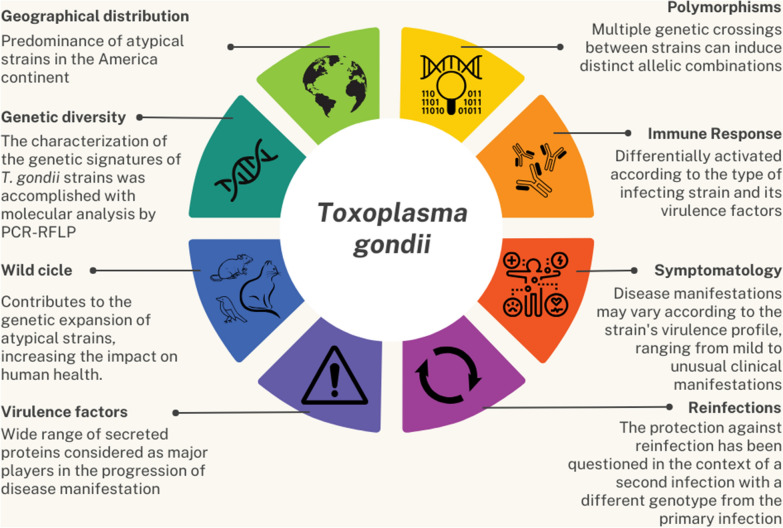

## Background

*Toxoplasma gondii* is an obligate intracellular protozoan parasite that is able to infect a wide range of warm-blooded animals, including humans [1, 2]. The prevalence of *T. gondii* infection in humans varies widely, and is as high as 90% in some areas of the world [3]. Toxoplasmosis outbreaks have been reported worldwide, with the Americas accounting for almost 74% of reported outbreaks in the past decades. Brazil, with 35% of reported outbreaks [4], is considered a hotspot for toxoplasmosis [5]. In addition to its worldwide distribution and highly diverse host range, *T. gondii* displays great genetic diversity, which is partly responsible for the variability in infection severity. *Toxoplasma gondii* strains are traditionally classified as ‘clonal’ or ‘atypical’, according to their genetic characteristics. Recent studies targeting microsatellite sequences showed that although so-called clonal strains share genotypic markers, they differ at the fingerprint level. Because they are not actual clones, the term “archetypal strains” has been proposed instead of clonal strains [6–8]. Thus, in this review we use the terms archetypal strains (types I, II and III genotypes) and non-archetypal strains (new strains, the identification of which is based on divergence in allelic distribution), instead of the terms clonal and atypical strains.

The archetypal strains belong to genotypes I, II, and III, which display less than 1% genetic divergence and are possibly derived from a single genetic crossing [9, 10]; these strains are widespread across the Northern Hemisphere, where they are mainly found in the USA and Europe. Howe and Sibley [9] showed that more than 95% of strains from Europe and North America could be classified as genotype I, II, or III. More recently, it was established that genotypes II and III are dominant in Europe and North America [11]. The diversity of *T. gondii* found in Europe and the Americas was recently assessed by new phylogenetic methods, which provided new insights into the evolution of *T. gondii* archetypal and non-archetypal lineages [12]. Recent molecular analysis revealed that the genetic diversity of *T. gondii* in Mexico is more similar to that observed in South America than in North America [13].

Novel *T. gondii* genotypes have been designated as atypical, exotic, recombinant, or non-archetypal, based on their allelic composition [14, 15]. An atypical strain has at least one atypical allele in one of the 11 genotyping markers. Strains with mixed profiles, with type I, II or III alleles at different markers, are termed recombinant strains.

Characterization of the genetic signatures of *T. gondii* archetypal strains is achieved by using the molecular technique polymerase chain reaction-restriction fragment length polymorphism (PCR–RFLP). The molecular techniques used to identify and characterize *T. gondii* genotypes have improved over time, accelerating the discovery of new genotypes [16, 17]. The ability to identify single nucleotide polymorphisms in *T. gondii* has enabled the identification of new strains, which also allows for a better picture of the parasite’s population genetics. Multilocus PCR–RFLP, microsatellite analysis, and multilocus sequence typing have improved the detection of genetic variations at strain level [16, 17], enabling refined characterization of non-archetypal strains, and elucidation of the population genetics and geographic distribution of *T. gondii.*

The diversity of *T. gondii* non-archetypal strains in South America is well recognized, with these strains differing significantly from those observed in the Northern Hemisphere [7, 11, 18]. An analysis of *T. gondii*’s geographical distribution undertaken almost a decade ago revealed a highly diverse population structure for strains in South America, with 156 distinct genotypes determined for the 646 strains analysed. Of these genotypes, 106 were found exclusively in Brazil [11]. Non-archetypal strains from South America significantly diverge from those of North America and Europe, with the haplogroups showing distinct geographical separation, and a varying number of specific polymorphisms [19].

Considering the great genetic diversity and widespread occurrence of *T. gondii* genotypes in South America, our review will focus on the main findings from that continent on the identification of non-archetypal strains and their interaction with the host immune system. We will also discuss virulence profiles associated with different genotypes of this parasite and their possible impact on the clinical outcomes of human toxoplasmosis.

## Genotyping of non-archetypal strains of *T. gondii*

A recent study [20] that summarized the occurrence of *T. gondii* strains in felids worldwide, and included data from North and South America, revealed a marked predominance (~ 74%) of non-archetypal strains in the American continent. In Central and South America, *T. gondii* non-archetypal strains are abundant in various animal species, and an extremely high genetic diversity has been indicated for this region [19]. Some of these strains are more prevalent and widely distributed in Brazil: BrI (*Toxoplasma* database (ToxoDB) #6), BrII (#11), BrIII (#8), and BrIV (#17) [21]. The high genetic diversity of *T. gondii* in Brazil may be associated with several factors, including the great diversity of its hosts there [22]. In this section, we will be covering the main published findings on the identification and genotyping of non-archetypal *T. gondii* strains in Brazil (Table 1).

**Table 1:** Overview of the distribution of *Toxoplasma gondii* isolates and genotypes in Brazil

*T. gondii* ID	Genotype ID (ToxoDB)	Genotyping method	Genotype classification	Isolate source	Localization (Brazilian state)	References
TgCkBr107–116, TgCkBr141–145	#6, #7, #25, #28, #29, #30, #70, #77, #96, #105	Nested PCR	11 Non-archetypal	Free-range chickens	Pará	[121]
TgCkBr146–164, TgOvBrRS1–4, TgPkSMBra	#1, #2, #10, #14, #17, #26, #27, #76, #87, #270, #301	Nested PCR, PCR–RFLP	1 Archetypal (II), 11 non-archetypal	Free-range chickens, sheep, pork	Rio Grande do Sul	[121–123]
TgCatBr38–62; TgCatBr64–84; TgCpBr1–36; TgOvBr1–13; TgShBr1–4; TgShBr16–15; TgGtBr1–7, 9–12	#1–#20, #111, #171, #307	Nested PCR; PCR–RFLP	2 Archetypal (II); 49 non-archetypal; 4 mixed	Cats, capybaras, sheep	São Paulo	[22–24, 124]
TgCkBr210–233, TgCatBrPE01–02	#2, #3, #59, #60, #61, #62, #146	PCR–RFLP	2 Archetypal (II and III), 5 non-archetypal	Free-range chickens, wild cats	Fernando de Noronha Island	[125, 126]
TgCkBr188–209	#16, #30, #32, #44, #60, #94–#98	PCR–RFLP	10 Non-archetypal, 1 mixed	Free-range chickens	Mato Grosso do Sul	[127]
TgPgBr06–16, TgShBr54, TgShBr124, TgShBr127, TgCkBr284–308, TgCatBr85–89	#1–#10, #13, #36, #122, #235	Nested PCR, PCR–RFLP	14 Non-archetypal, 5 mixed	Pigs, sheep and free-range chickens, cats	Bahia	[28, 128–130]
TgCTBr1–27, TgWildBrMG1–6	#8, #11, #13, #36, #41, #67, #108, #162, #206–#212	PCR–RFLP	18 Non-archetypal, 1 mixed	Humans, wild birds	Minas Gerais	[25, 37]
TgPgBrRN1–5; TgCkBrRN1–13; TgGtBrRN1; TgGtBr8, 10	#1–#16, #109, #116, #163	PCR–RFLP	8 Non-archetypal	Sheep, goats, pigs and free-range chickens	Rio Grande do Norte	[24, 29]
TgCkBr234–250, TgCkBr252–265, TgCkBr267–274, TgCkBr277–281	#1, #6, #14, #65, #75, #108, #109, #162, #206, #213–#215	PCR–RFLP	11 Non-archetypal	Free-range chickens	Espírito Santo	[131]
TgCTBral, TgCTBrac, TgCTBrv, TgCtBRca, TgCkBrPr1–18	#6, #19, #21, #111, #152, #166, #175, #248, #251–#253	Nested PCR, PCR–RFLP	12 Non-archetypal	Humans, free-range chickens	Paraná	[132, 133]
TgHoFBr1, TgMWBr1, TgOncBr1, TgNbaBr1–3, TgCantBr1–3, TgWlpBr1–3, TgSpPBr2, TgLWpBr1, TgSbaBr2	#6, #11, #175, #195, #196, #231–#239, #241, #246	PCR–RFLP	17 Non-archetypal	Wild animals	Mato Grosso, Minas Gerais, Pará, Pernambuco and São Paulo	[134]
TgCkBrMA1–5	#06, #07, #109	PCR–RFLP	4 Non-archetypal	Free-range chickens	Maranhão	[135]
TgPgBRPB3, 8–14, 16–18, 20–24, 26, 27	#8, #13, #109, #114, #116, #203, #272–#277	PCR–RFLP	11 Non-archetypal, 1 mixed	Pigs	Paraíba	[136]
TgPgBrPI1–16, TgGtBrPI1–9	#7, #8, #13, #33, #57, #109, #116, #146, #163, #203	PCR–RFLP	11 Non-archetypal	Pigs and goats	Piauí	[34]
TgCkBrSC1–4	#10, #26, #53, #278	PCR–RFLP	1 Archetypal (I), 3 non-archetypal	Free-range chickens	Santa Catarina	[137]
TgCkAl1–2, TgCTBrAL1	#146, #162, #279	PCR–RFLP	3 Non-archetypal	Free-range chickens, human	Alagoas	[35, 138]
TgDgBrMT1–9, TgCatBrMT1, TgPwBrMT1–2, TgCefBrMT1–3, TgBfcuBrMT1, TgAagBrMT1, TgCpBrMT1–2, TgOceBrMT1, TgRhaBrMT1, TgCantBrMT1, TgMduBrMT1, TgLonloBrMT1, TgSAcoBrMT1, TgCpeBrMT1, TgCkBrMT1–51	#2, #6, #8, #11, #14, #19, #41, #99, #108, #109, #116, #140, #166, #190, #310, #311, #313–#317	PCR–RFLP	1 Archetypal (III), 21 non-archetypal, 6 mixed	Domestic animals, wild animals, humans, free-range chickens	Mato Grosso	[26, 27]
TgCkBrRj2–4, 6, 15, 21, 22, 28; TgCkBr10, 11, 13, 37, 59, 89; TgDgBr6; TgCatBr1	#6, #8, #11, #36, #51, #63, #65, #107	PCR–RFLP	17 Non-archetypal	Free-range chickens	Rio de Janeiro	[36]
TgCkBrGO1–15	#65	PCR–RFLP	1 Non-archetypal	Free-range chickens	Goiás	[139]

The genotyping analyses were performed using SAG1, SAG2 (5′3′SAG2 and/or alt.SAG2), SAG3, BTUB, dense granule protein 6 (GRA6), c22-8, c29-2, L358, Apico and CS3 loc as the molecular targets

*ToxoDB* *Toxoplasma* database, *PCR–RFLP* polymerase chain reaction-restriction fragment length polymorphism

Mixed genotypes, which indicate the simultaneous presence of two strains in the same host, have been identified in free-range chickens, goats, capybaras, cats, a newborn child [21, 23–29], and in other animals [30–32]. Different strains coexist dynamically in nature and the presence of mixed genotypes of *T. gondii* in intermediate hosts may facilitate the genetic recombination of the parasite in the gut of a definitive host upon its ingestion of distinct strains [16]. Three main hypotheses have been proposed to explain the existence of mixed infections in a definitive host. First, a mixed infection could be due to an impaired immune response against the superinfecting genotype, allowing the second (or third) genotype to establish a chronic infection. Secondly, the feeding behaviour of felids, which can involve simultaneously (or within a short period of time) preying on various intermediate hosts, offers another opportunity for mixed infections to occur. Thirdly, the ingestion of oocysts containing mixed progeny resulting from a sexual outcross could be a mechanism by which a mixed infection may be introduced into a definitive host. These hypotheses, if supported, could collectively shed light on the intriguing dynamics of mixed infections in the life cycle of *T. gondii* [33].

Carneiro et al. [25] identified 25 strains, all with non-archetypal genotypes, from the peripheral blood of newborns with congenital toxoplasmosis in the state of Minas Gerais, southeastern Brazil (Table 1). Only two of them were considered avirulent (ToxoDB #8/BrIII, #207), while the others were virulent or of intermediate virulence in experimentally infected mice. The fact that the mouse-virulent strains were isolated from newborns with severe toxoplasmosis and the avirulent strains from asymptomatic children may indicate that there is some correspondence between virulence in mice and disease in humans [25].

In the Central-West region of Brazil there is also a high genetic diversity of *T. gondii*, and different strains have been isolated from domestic and wild animals, and from humans. A recent study [26] examined 22 strains obtained from nine dogs, one cat, 10 wild animals, and two women in the state of Mato Grosso/Brazil. Multilocus PCR–RFLP revealed 11* T. gondii* RFLP genotypes (Table 1), nine of which had been previously described, and two of which were new [26]. More recently, the same authors isolated 51* T. gondii* strains from free-range chickens in the state of Mato Grosso [27]. Fifty of the strains were completely genotyped, and were shown to comprise 17 previously described non-archetypal genotypes and five new genotypes. In addition, mixed infections were observed in five of the free-range chickens. In sum, these studies confirm the great diversity of *T. gondii* strains in animals and humans in Brazil.

Deiró et al. [28] isolated non-archetypal strains of *T. gondii* from seven naturally infected cats in Bahia, in the Northeast region of Brazil. PCR–RFLP with 11 genetic markers revealed four distinct genotypes. In the genotyping analysis using microsatellite markers, five non-archetypal genotypes were detected in the cats [28].

The occurrence of non-archetypal genotypes in animals slaughtered for human consumption has also been reported in Brazil. Clementino-Andrade et al. [29] identified 19 T*. gondii* isolates from animals intended for human consumption from the state of Rio Grande do Norte, in the Northeast region of Brazil, of which 17 were classified as virulent based on mice bioassays (ToxoDB #1, #13, #109, #116, #163). Four distinct genotypes were found, including two new ones identified via PCR–RFLP. Rêgo et al. [34] isolated non-archetypal strains of *T. gondii* from swine and goats raised and slaughtered in the state of Piauí, Northeast region, Brazil. Eleven different genotypes were identified using PCR–RFLP, including a unique one, detected in swine, that had not been previously reported for any other host (Table 1). The 22 other strains belonged to 10 genotypes. In contrast to observations from other states in Brazil, where many of the *T. gondii* strains examined were reported to be virulent for mice, 72% of the isolates obtained in Piauí were avirulent. Four (16%) displayed intermediate virulence and three (12%) were virulent for mice (ToxoDB #7, #33, #163) [34].

In another study from northeastern Brazil, Santos Silva et al. [35] demonstrated the occurrence of anti-*T. gondii* antibodies in free-range chickens that were destined for human consumption. Out of 200 blood samples, 72 (36%) were considered positive by indirect immunofluorescence. Two *T. gondii* strains were isolated, both of which were characterized as non-archetypal and classified as genotypes #146 and #279, with the latter being a new genotype [35].

Casartelli-Alves et al. [36] characterized *T. gondii* strains obtained from free-range chickens reared in the state of Rio de Janeiro, Brazil. Seventeen non-archetypal genotypes were identified among the 36* T. gondii* strains obtained, of which eight had been previously described for different hosts and nine were described for the first time [36].

Non-archetypal genotypes have also been detected in wild animals from Brazil. Rêgo et al. [37] obtained six *T. gondii* isolates from wild free-range birds rescued in the state of Minas Gerais (Table 1). Five different genotypes were identified by PCR–RFLP, including a new genotype. Of those, three isolates were classified as having intermediate virulence and three as avirulent (ToxoDB #1, #8, #13) for mice. Non-archetypal genotypes detected in free-living wild birds have also been detected in humans, domestic animals, and animals slaughtered for human consumption [37].

*Toxoplasma gondii* is widespread in South America, and in addition to Brazil, non-archetypal genotypes have been reported in Colombia [38] and Argentina [39, 40]. Pérez-Grisales et al. [44] reviewed 8103 reports of *T. gondii* in Colombia, of which 86% were from humans and 82% from urban areas. Moré et al. [39] isolated and genotyped *T. gondii* from serologically positive free-range chickens in Argentina [41]. On one of the farms, they found a non-archetypal genotype that had been previously obtained from chickens in Brazil [42]. They also found the isolate TgCkN21Arg, on another farm, which showed a RFLP pattern identical to that of the Brazilian avirulent archetypal BrIII which had been reported from other species of animals [22–24]. In Argentina, non-archetypal *T. gondii* genotypes have also been reported as the cause of congenital human toxoplasmosis [40]. Six isolates obtained from umbilical cord blood and placenta were characterized as non-archetypal strains, classified into five genotypes. Of the five genotypes identified from the congenital infection, two had not been previously reported [40], whereas three had been described (ToxoDB #14, #138, and #182) [42, 43]. In sum, these findings indicate the widespread circulation of non-archetypal genotypes in different hosts and geographic regions of South America.

The high diversity of *T. gondii* strains circulating in Brazil is noteworthy. The identification of both avirulent and virulent strains in animals intended for human consumption underscores the potential health risks of *T. gondii*, and the need for better monitoring of the food supply chain. Additionally, the detection of non-archetypal genotypes in wild animals, poultry and other birds, and in humans, suggests that they have a wide distribution among various host species. In addition, as many of the studies discussed above were conducted in limited geographical areas and focused on specific host species, the diversity of *T. gondii* in South America may actually be underestimated. A comprehensive understanding of the genetic diversity of *T. gondii*, the clinical implications of toxoplasmosis and its potential risk for public health, require more interdisciplinary research and surveillance efforts.

## Immunological response to *T. gondii* infection

The immune response to *T. gondii* infection has been extensively studied in experimental murine models. Mice Toll-like receptor 11 (TLR11) recognizes *T. gondii* profilin and, together with TLR12 [44], triggers interleukin (IL)-12 and interferon (IFN)-γ production via the myeloid differentiation primary response 88 protein pathway, the activation of which is essential to elicit an efficient Th1 response against the parasite [45–48].

The innate production of IL-12 and IFN-γ drives the differentiation of Th1 lymphocytes, acting as a positive feedback loop to enhance the inflammatory response and its capacity to control parasite replication [49, 50]. Although the capacity of *T. gondii* to elicit an efficient IFN-γ-mediated inflammatory response is well established, distinct strains may induce different types of immune responses [51]. In this context, the genotypic characteristics of *T. gondii* strains, and the differences among them, can determine infection outcomes, based on their ability to up- or downregulate the inflammatory response.

## *Toxoplasma gondii* archetypal strains: virulence factors and immunological activation

*Toxoplasma gondii* archetypal strains display distinct levels of virulence [52, 53]. In addition, these strains may activate the immune response differently, mainly through the action of rhoptry protein 5 and dense granule-secreted proteins (Table 2), which display significant genetic variation [54].

**Table 2:** The main proteins of *Toxoplasma gondii* identified as virulence factors and their interaction with the immune system

Proteins	Source	Virulence mechanisms and immunological importance	References
Rhoptry protein 5 (ROP5)	Rhoptries	Regulation of ROP18 catalytic activity, forming a ROP5/ROP18 complex Blocking of interferon (IFN)-γ-mediated macrophage microbicidal activity Limits antigen presentation in macrophages and dendritic cells	[58, 140]
ROP7		Interacts with the NACHT domain of NLR family pyrin domain-containing 3 (NLRP3) and promotes inflammasome hyperactivation and induction of interleukin (IL)-1β	[66]
ROP16		Phosphorylation of signal transducer and activator of transcription protein 3 (STAT3) and STAT6 and induction of IL-12 and IFN-γ-mediated inflammatory response	[57, 141]
ROP18		Downregulation of the macrophage production of IL-16, TNF-α and IL-12 by phosphorylation of p65 domain of nuclear factor kappa B (NF-κB) Suppression of antigen presentation mechanisms in macrophages and dendritic cells	[57, 142]
GRA3	Dense granules	Regulation of the antigen presentation mechanisms in an IFN-γ or cell-independent manner	[57]
GRA6		Regulation of the activation of nuclear factor of activated T cells 4 (NFAT4) in a strain-specific manner, modulating the expression of C-X-C motif chemokine ligand 2 (*CXCL2*) and C-C motif chemokine ligand 2 (*CCL2*) and the recruitment of neutrophils	[76]
GRA7		Interacts with the ROP5/ROP18 complex, binding directly to ROP5 to promote the phosphorylation of immune-regulated guanosine triphosphatase 6 (IRG6) and inactivation of IRG-mediated resistance system	[73]
GRA12		Confers resistance to IFN-γ-mediated immune response, leading to better parasite survival and establishment of infection in a strain-specific manner	[68, 74]
GRA15		Activation of NF-κB via TNF receptor-associated protein 6 (TRAF6) Induction of the inflammasome activation pathway and contributes to IL-1β, IL-12 and TNF-α production	[52, 67, 69]
GRA24		Act as an activator of the immune response, modulating IL-12, IL-1β and TNF-α production via mitogen-activated protein kinase (MAPK) activation and the NF-κB pathway, independently of MYD88 innate immune signal transduction adaptor	[52, 75]
GRA25		Modulation of CCL2 production by macrophages and contributes to efficient parasite survival	[72]

Secreted rhoptry proteins such as rhoptry protein 16 (ROP16) and ROP18 act as virulence factors in murine models [55, 56]. These proteins have distinct functions depending on the stage of infection. ROP16 is capable of interfering with the signal transducer and activator of transcription protein 3 (STAT3) and STAT6 signalling pathway, leading to the suppression of IL-12 production. ROP18 can suppress antigen presentation in macrophage and dendritic cells of mice, in both an immune-regulated guanosine triphosphatase (IRG)-independent and dependent manner [57, 58]. IRGs are a family of GTPases that play a crucial role in host defence against intracellular pathogens, including *T. gondii*. These proteins are activated by IFN-γ [59]. Selective recruitment of IRGs to the nascent parasitophorous vacuole membrane (PVM) of susceptible strains leads to destabilization of the vacuole, release of the parasite into the cytoplasm and eventual parasite death in mouse cells [60, 61]. Similar to ROP18, ROP17 targets a common pathway by phosphorylating a member of the IRG family that shows an overlapping function [62]. The phosphorylation ability of ROP18-ROP17 depends on the presence of virulent alleles of pseudokinase ROP5 [63, 64]. In type I parasites, the combination of highly expressed ROP18 and the virulence enhancing ROP5 locus makes the triad of ROP5ROP18ROP17 virulence effectors most potent. Conversely, type II strains exhibit an active ROP18 but an avirulent* ROP5* locus. Notably, type III strains, despite possessing the virulence-enhancing* ROP5* locus, are entirely avirulent unless highly expressed ROP18 is present [65].

Another important virulence factor is the pseudokinase ROP5, which is particularly significant for the type I strains. The deletion of the *ROP5* locus leads to loss of virulence [63]. The virulence profile observed for types I and II (type I is considered to be virulent and type II to be avirulent) can be at least partially explained by the polymorphisms in amino acid sequences from *ROP5* [63, 64].

Besides their impacts on mechanisms of antigen-presentation, ROP7, dense granule protein 15 (GRA15), and *T. gondii* matrix antigen 1 are capable of interfering in the activation of the inflammasome pathway. ROP7 can act as a second signal in the activation of NLR family pyrin domain-containing 3 (NLRP3) inflammasome, and interact with the NACHT domain of NLRP3, stimulating IL-1β and leading to inflammasome hyperactivation [66]. GRA15 and *T. gondii* matrix antigen 1, both of which belong to the group of proteins produced by the dense granules, are responsible for maintaining a fine balance between activation and dampening of inflammasome activation, respectively [67].

In addition to its role in inducing innate immune responses through the inflammasome pathway, GRA15 has the ability to stimulate IFN-β production via the cGAS/STING pathway [68, 69]. The activation of this signalling cascade enables infected mice to survive infection, as it confers resistance to infection through the control of parasite replication. This is supported by studies showing that mice infected with GRA15 knockout parasites had reduced levels of cytokines such as IFN-β, CXCL10, IFN-γ, and IL-12, followed by higher parasite burden and mortality [68]. Furthermore, GRA15 from type II strains efficiently induces nuclear factor kappa B (NF-κB) activation and its nuclear translocation, contributing to the induction of IFN-γ [70]. It is important to note that there are differences in the parasite-released proteins between strains of the same genotype. For instance, the *T. gondii* type I RH strain lacks the ability to effectively activate NF-κB due to defective expression of GRA15, leading to lower levels of secreted IL-12. In contrast, the type I GT1 strain can efficiently activate NF-κB, inducing higher amounts of IL-12, by encoding a functional GRA15 [71].

Other GRAs, such as GRA6, GRA7, GRA12, GRA24, and GRA25, interfere in the induction of immune responses, favouring the parasite’s replication and the establishment of the chronic infection stage [72–75]. The activity of these proteins may vary and they can function as important virulence factors, as shown by the significantly reduced mortality observed in mice infected with *T. gondii* types I and II strains after GRA12 deletion [68]. Furthermore, the protein GRA6 is an important factor in the establishment of strain-specific immunity against *T. gondii.* Through its ability to induce the activation of NFAT4, and consequently the production of chemokines involved in the recruitment of inflammatory cells such as neutrophils, GRA6 plays a major role in determining the differences in virulence observed among the archetypal genotypes I and II [76].

The activities of strain-specific secreted proteins of *T. gondii* can explain differences in the severity of infection by known archetypal genotypes, in that these proteins can trigger an early or delayed immune response and interfere in the cell recruitment mechanisms responsible for the parasite’s control [18, 77]. In human infection, genetic variations among *T. gondii* strains are associated with different levels of disease severity. Highly diverse genotypes of *T. gondii* were found to be associated with distinct virulence profiles in strains that were mainly from South America [78]. Other geographic differences in disease severity, such as severe ocular toxoplasmosis and increased occurrence of severe congenital toxoplasmosis in areas with a high incidence of non-archetypal strains, have also been shown [79, 80].

While the above-mentioned studies shed light on virulence factors and the immunomodulation mechanisms employed by different *T. gondii* strains, these mechanisms are complex and multifaceted. For instance, the interactions between parasite-secreted proteins and the host immune response are not fully understood. Additional research is needed to clarify details of these interactions and how they may contribute to disease severity. Despite numerous published studies characterizing the nature of non-archetypal *T. gondii* strains in South America, few experimental studies have compared the infection dynamics and pathogenicity of these strains with the archetypal strains. Furthermore, there has been limited investigation into the presence of polymorphisms in key virulence factors of non-archetypal *T. gondii* strains and their immune-modulatory capabilities. Closing these knowledge gaps will be instrumental in advancing our understanding of the biology and pathogenesis of *T. gondii*.

## *Toxoplasma gondii* non-archetypal strains: virulence factors, immunological activation, and disease outcomes

Secreted proteins described for the *T. gondii* archetypal strains have also been implicated as virulence factors for non-archetypal strains in mice models [81, 82]. Differences in the virulence of *T. gondii* non-archetypal strains are also related to polymorphisms in their secreted proteins, such as GRAs and ROPs, which results in their distinct abilities to induce an immune response [80, 83]. Besides the polymorphic nature of ROPs and GRAs, their ability to form protein complexes is key to the prediction of virulence among *T. gondii* non-archetypal strains. The importance of ROP18 and ROP5 to the virulence of archetypal strains, and that of specific allelic combinations of these two proteins to the virulence of non-archetypal strains in murine models, is acknowledged [84]. Shwab et al. [84] confirmed that the *ROP18* type 1 allele (representing a type I archetype) is linked to virulence, while the *ROP18* type 2 and 3 alleles (representing type II and III archetypes, respectively) are associated with avirulence. Additionally, they observed that when allele 3 (*ROP5*) is combined with allele 1 (*ROP18*), the phenotype is virulent, whereas when combined with allele 3 (*ROP18*), the phenotype is avirulent [84].

Rego et al. [34] showed that the virulence profile of *T. gondii* strains obtained from pigs in Brazil was related to the presence of a specific allelic combination of *ROP18* and *ROP5* (allele 4 for *ROP18* and allele 3 for *ROP5*). Furthermore, virulent *T. gondii* strains with the same allelic combinations for these proteins were able to induce higher levels of inflammatory cytokines, such as IFN-γ, IL-12, IL-17, and TNF-α, during early infection in mice [85]. However, the allelic profile of *ROP5* and *ROP18* can change among strains considered to be avirulent or to have intermediate virulence in mice [86].

In a study conducted in Argentina [86], a combination of *ROP18* and *ROP5* allele types that could be used to predict virulence was detected in a *T. gondii* strain that was non-lethal to mice. In another study [87], virulent Caribbean and Brazilian strains harboured distinct *ROP18* and *ROP5* allele types (alleles 3 and 1 for the Caribbean strain and allele 1 and 3 for the Brazilian strain for *ROP18* and *ROP5*, respectively), while avirulent Caribbean and European strains shared the same allelic combination (allele 2 for both loci). The difference in virulence between these distinct *T. gondii* strains which shared (or did not share) the same combination of* ROP18* and* ROP5* allele types might have been due to secondary factors that are involved in the parasite’s ability to induce pathology in mice and humans.

The virulence profiles of a diversity of genotypes in mice may be indicative of the severity of toxoplasmosis they cause in humans. *Toxoplasma gondii* non-archetypal strains obtained from human cases of congenital toxoplasmosis with different levels of severity were able to induce different pathologies in mice, even when the strains were of the same genotype [88]. This suggests that although the classification of genotypes can be helpful in elucidating aspects of their epidemiology and population genetics, it may not fully reflect the biological diversity among strains. Human infections by highly virulent non-archetypal strains can be characterized by differences in their inflammatory stimuli compared to infection caused by archetypal strains. For instance, Colombian patients with ocular toxoplasmosis who were infected by highly heterogeneous strains showed a suppressed IFN-γ response although high levels of TNF-α were induced [89]. The ability of certain strains to downregulate IFN-γ contributes to the host’s reduced capacity to control the parasite’s replication, favouring its dissemination and contributing to tissue damage due to high levels of TNF-α. Thus, the unbalanced immune response triggered by virulent non-archetypal strains may contribute to the development of severe toxoplasmosis. In our recent study [90] comparing chronic murine infection caused by a type II (ME-49) strain and a non-archetypal (CK-2) strain (ToxoDB #163), the non-archetypal strain induced higher levels of IFN-γ and TNF-α in brain tissue compared to the archetypal strain. Additionally, our study revealed that infection with the non-archetypal strain altered the levels of neuroinflammatory mediators and led to the development of behaviour indicative of depression and anxiety, indicating a potential link between persistent inflammation and behavioural changes. For years, serological data have indicated a potential connection between prior *T. gondii* infection and various psychiatric disorders, including depression [91], schizophrenia [92, 93], bipolar disorders [94], attempted suicide [95] and other conditions [96–98]. It is thus crucial that further research is undertaken to improve our understanding of the mechanisms underlying this potential link. Experimental models can provide valuable insight into the relationship between *T. gondii* infection and psychiatric disorders, by shedding light on the underlying biological processes. It is also essential to determine if these relationships hold true for human populations. Moreover, clinical studies exploring associations between *T. gondii* and psychiatric disorders in humans should also examine how genetic variation in the parasite affects the observed outcomes, as this could help to clarify the likely intricate relationship between *T. gondii* infection and psychiatric disorders.

Although there is little knowledge regarding the exact pathways triggered by the non-archetypal strains, studies have demonstrated the ability of *T. gondii* secreted factors to induce the inflammatory response. Melo et al. [99] showed that GRA15 from non-archetypal strains can induce the production of IFN-β by macrophages and trigger NF-κB activation, regardless of the allelic composition. Genetic variations among *T. gondii* non-archetypal strains of the same genotype can drive different immune responses, directly impacting the course of infection in the host. Non-archetypal strains with the same genotype can indeed trigger different immune responses by interacting with different components involved in macrophage activation, and avirulent strain was able to induce alternative activation of macrophages by phosphorylation of STAT6. However, a less virulent strain drove classic macrophage activation by inducing NF-κB activation and IL-12 and TNF-α production [100].

It is assumed that different immunological activation pathways may be involved in disease outcomes of infections with non-archetypal strains of *T. gondii* with distinct virulence profiles. A study performed in France identified a non-archetypal strain as the causative agent of severe toxoplasmosis in an immunocompetent patient, with lung involvement and the presence of tachyzoites in the bronchoalveolar lavage fluid [101]. Non-archetypal strains have also been identified in cases of congenital toxoplasmosis, and were associated with severe infection [102]. In Egypt, *T. gondii* seropositivity was found to be significantly higher in children with hydrocephalus and epilepsy; the presence of non-archetypal strains was confirmed by PCR–RFLP [103]. The strain should also be taken into consideration as a potentially important factor in the case of pregnant women, as it may have an impact on fetus survival, as well as on the severity of a newborn’s ocular and/or cerebral impairment [104].

Importantly, the long-held assumption that a previous infection with *T. gondii* protects the host from subsequent reinfection due to immune memory is now under discussion, with reports of reinfection in humans [105–110] and experimental models [111–113], especially where the second infection is with a different genotype from the primary infection [105, 110, 112–115], as reported for an experimental murine model [116]. In this context, the development of a point-of-care diagnostic test that could distinguish primary from secondary infections would be of value for the monitoring of pregnant women. It is also important to include reinfection as a possible cause in clinical guidelines, with specific prophylactic guidance for pregnant women who are IgG− or IgG+.

Another important matter is the occurrence of a wild cycle of *T. gondii*, which contributes to the diversity of genotypes that can infect humans, with possibly severe disease manifestations in both immunosuppressed and immunocompetent patients. Severe and unusual cases of toxoplasmosis were reported in Amazonia and named “Amazonian toxoplasmosis” [117, 118]. Demar et al. [119] reported cases of acute toxoplasmosis with visceral complications caused by a single non-archetypal strain (with a unique multilocus genotype) with a high virulence profile in the Amazonian country Suriname, which borders French Guiana and Guyana. In this outbreak, immunocompetent patients presented with multiple organ failure, including signs of respiratory distress, renal insufficiency, and hepatic impairment; there were also some cases of lethal congenital toxoplasmosis [119]. Another, more recent, Amazonian toxoplasmosis outbreak was recorded in French Guiana, in which 37% of 54 patients had confirmed acute toxoplasmosis [120]. The main symptoms were fever, cough, and complications such as hepatic cytolysis. A foetal death caused by a strain belonging to the Amazonian genetic group was also recorded during this outbreak [120].

The wild cycle of *T. gondii,* which contributes to the expansion of non-archetypal isolates, may increase the impact of toxoplasmosis on public health, regardless of the immunological status of patients. Furthermore, the identification of severe and unusual cases of toxoplasmosis due to infection with highly virulent non-archetypal strains is of great importance for the monitoring of risk factors in transmission areas and possibly for the prediction of outbreaks.

The clinical evolution of toxoplasmosis in adults and children, and in the congenital form, is not completely understood. Nevertheless, it is evident that the host immune response and the diversity of parasite strains are pivotal to the clinical evolution of this disease. The complex interplay between these factors is also influenced by individual host factors, including genetic predisposition and variation in immune environments, which influence disease progression. Due to ongoing advances in molecular genotyping, new *T. gondii* isolates and genotypes are being constantly identified. This highlights the urgent need for a comprehensive understanding of the impact of strain variability on immune response activation, congenital transmission rates and disease outcomes. Genetic variation in *T. gondii* may also have implications for treatment success and vaccine development. Investigating these relationships is pivotal to identifying possible patterns of immune response regulation, drug susceptibility, and pathogenicity.

## Conclusions

This review highlights the complexity and wide genetic diversity of *T. gondii*, one of the consequences of which is the classification of its strains into archetypal and non-archetypal types. Despite a growing number of studies that validate the existence of great genotypic diversity among *T. gondii* strains in South America, where non-archetypal strains significantly differ from those found in North America and Europe, few studies have attempted to further investigate the virulence factors and immunological mechanisms related to infection with non-archetypal strains. This review highlights the intricate relationship between strains of *T. gondii* and the host immune response, and sheds light on the factors that contribute to the variability in infection severity. It has become increasingly clear that the virulence of these strains is strongly influenced by specific proteins that they secrete, and particularly those secreted by the rhoptry and dense granule organelles, which have distinct roles during infection. Understanding how strain-specific interactions between *T. gondii* and the host immune system influence infection severity is vital, given the parasite's worldwide distribution and genetic diversity. This knowledge should aid the development of more targeted strategies for the diagnosis, treatment, and prevention of toxoplasmosis, particularly in regions with unique strain profiles. Furthermore, this review shows that *T. gondii* strains, even within the same genotype, can induce different immune responses and clinical manifestations in both mice models and humans. Variation in the activation of immune pathways may explain the diverse clinical presentation of patients infected with different strains. Geographic differences in strain diversity, such as the emergence of highly virulent non-archetypal strains in Amazonia, are of significance for public health. These highly virulent non-archetypal strains can lead to severe and unusual cases of toxoplasmosis, in both immunosuppressed and immunocompetent individuals. An ability to recognize variation in the disease is thus crucial for the monitoring of transmission risk factors and the prediction of potential outbreaks. Finally, the high heterogeneity of *T. gondii* strains, particularly in South America, highlights the complexity of toxoplasmosis. Advancements in molecular genotyping have led to the identification of numerous isolates and genotypes, which contribute to unique manifestations of the disease. Understanding the immune responses and inflammatory pathways induced by these diverse strains is essential for predicting disease outcomes and developing effective diagnostic and preventive strategies.

## Data Availability

This review is based on published, publicly available data, and did not involve the collection of new data.

## Abbreviations

GRA: Dense granule protein
IFN: Interferon
IL: Interleukin
IRG: Immune-regulated guanosine triphosphatase
NF-κB: Nuclear factor kappa B
ROP: Rhoptry protein
ToxoDB: *Toxoplasma* database
